# Growth, yield, and yield variables of onion (*Allium Cepa* L.) varieties as influenced by plantspacing at DambiDollo, Western Ethiopia

**DOI:** 10.1038/s41598-022-24993-x

**Published:** 2022-11-29

**Authors:** Diribsa Alemu, Chala Kitila, Weyessa Garedew, LetaTesfaye Jule, Bayissa Badassa, N. Nagaprasad, Venkatesh Seenivasan, Abel Saka, Krishnaraj Ramaswamy

**Affiliations:** 1 Department of Plant Sciences, College of Agriculture and Veterinary Medicine, DambiDollo University, Dambi Dollo, Ethiopia; 2 Department of Horticulture and Plant Sciences, College of Agriculture and Veterinary Medicine, Jimma University, Jimma, Ethiopia; 3 Physics Department, College of Natural and Computational Sciences, Dambi Dollo University, Dambi Dollo, Ethiopia; 4 Centre for Excellence in Technology Transfer and Incubation, Dambi Dollo University, Dambi Dollo, Ethiopia; 5 Ministry of Innovation and Technology, Addis Ababa, Ethiopia; 6 Department of Mechanical Engineering, ULTRA College of Engineering and Technology, Madurai, 625 104 Tamil Nadu India; 7 Department of Mechanical Engineering, Sri Eshwar College of Engineering, Coimbatore, India; 8 Mechanical Engineering Department, College of Engineering Science, Dambi Dollo University, Dambi Dollo, Ethiopia

**Keywords:** Biological techniques, Biotechnology, Plant sciences, Engineering

## Abstract

Onion (*Allium cepa* L.) is an important bulb plant grown worldwide. Proper use of the agronomic practice has undoubtedly contributed to growing crop yields. The right level of any farming practice, like the distance between plants, plant density, date of planting, and time of harvest, can produce the wanted outcomes. Therefore, this research was piloted to evaluate the influence of plant spacing on the development of bulb harvest-related traits of onion varieties in Dambi Dollo University, Western Ethiopia, in 2021. Three onion varieties (Adama red, Monarch, Nafis) and four intra-row spaces (6 cm, 8 cm, 10 cm, and 12 cm) in factorial combinations were settled by a complete randomized block design which was simulated three times. The findings of the study indicate that all the factors related to crop growth and bulb yield of onion varieties were mainly influenced by different kinds and plant spacing. Conversely, the collaboration of these two factors did not affect all other factors, but the interaction of the two factors had a great effect on the days to maturity measured in this study. The highest plant height was registered on the onion planted at a distance of 10 cm (59.83 cm) and 12 cm (59.08 cm) distance between plants. The high commercial yields (34.44 t ha^−1^) and entire bulb yield (35.40 t ha^−1^) were found in the Nafis variety. The highest marketable yields (31.12 ha^−1^) and entire marketable yield (31.78 ha^−1^) were recorded on an onion plant planted 10 cm between plants. Therefore, in the research area, farmers can use a variety of Nafis and a 10 cm distance between plants to increase their onion production.

## Introduction

Onion (*Allium-cepa*L.) belongs to the Alliaceae family and the genus *Allium* ^1^, and it is an important crop for bulb plants grown globally. Onions are cultivated for use in a green state and mature bulbs, and some diversity is observed in the eastern Mediterranean countries, which are the main basis of onion genetic variability, as well as they are supposed to be native to onion ^2^. Usually, onion is a plant of open, sunny, dry areas, but most species are initiated in cliffs, on waterless foothill slopes, in stony or rocky open areas, or in dry, open, flooded summer plants ^1^.

It is essential in the Ethiopian diet daily, which is widely consumed as a spice and or vegetable in strews ^3^, and it is the main foundation of flavonoids in human food and the use used to reduce the danger of cancers, temperament diseases, as well as diabetes. The quality parameter of onion as a percentage of single bulbs is essential to encounter the needs of both fresh and processing market buyers ^4^.

In Ethiopia, onions are an important economic center due to their easiness of cultivation, more yield per hectare, and the irrigation system increasing onion production from time to time. In Ethiopia, the total area under onion production was about 38,952.58 ha, of which 3,460,480.88 tons were produced in 2020/2021, with an average yield of about 8.8 t ha^−1^ (CSA, 2021). This showed that the production of onion in Ethiopia (8.8 t ha^−1^) is significantly under the global average (18.8 t ha^−1^). Production of onions can be constrained by unsuitable spacing, deprived fertilization, and inaccessibility of quality planting materials organized with other cultural practices (CSA, 2021).

In Ethiopia, five different kinds of onion (Adama Red, Bombay Red, Red Creole, Melkam, and Nasik Red (Dereselegn) have been released by the research center. The varieties are broadly grown in Ethiopia and Awash Valley, and Lake Region is Bombay Red and Adama Red in larger quantities. However, the adaptive capacity of these varieties is not known in the KellemWollega zone. Proper use of the cultural practice has undoubtedly contributed to growing crop yields. The right farming practice, like planning distance, plant density, and date of planting, time of harvesting, can give the wanted outcomes. The use of recommended plant spacing has two advantages. It evades robust competition among plants through the development influences like marine, nutrients, and sunlight. In contrast, an optimal number of plants allows for the effective use of accessible cropland without waste ^6^. In order to improve onion production, information on the appropriate production package is required ^5,6^.

Appropriate plant spacing allows farmers to retain suitable plant densities in the field. Consequently, it can evade too much or too little in an area that has deleterious influences on the development and profit of onion. The nationally recommended distance between onion plants was 10 cm, grounded on the study conducted in the central rift valley of the country about years ago ^6^.

However, producers are complaining about the 10 cm distance between plants producing large bulb sizes that a consumer can choose for home use ^7^. As a result, in the real world, the practice accepted by farmers is smaller and/or wider than recommended. Growers in the KellemWollega Zone do not use recommended crop varieties and improved varieties to produce onions, and no new varieties have been introduced locally. In addition, a small amount of research was conducted in the study area to optimize plant spacing and identify better-performing varieties to date. Therefore, onions grown in the study area do not meet the needs of the local market use and people in the study area are forced to buy onions produced in the central part of Ethiopia. Therefore, the present study is designed to identify optimum intra-row spacing that maximizes the onion yield and to evaluate the adaptability of different onion varieties for yield and yield-related trials in the DambiDollo area.

## Materials and methodology

### Explanation of the study area

In 2021, research field testing was conducted at the research site of Dambi Dollo University under rain-fed conditions. The pilot investigation was successful. In the western region of Oromia, Ethiopia, in the Kellem Wollega Zone, the research site may be found at an elevation of between 1500 and 1740 m above mean sea level. This location is approximately 652 km distant from Addis Ababa in the westward direction. Annual precipitation averages between 850 and 1200 mm in this region. Temperatures in the region range from a low of 15 degrees Celsius to a high of 28 °C. The soil used in the study was classified as sandy loam^8^.

### Descriptions of experimental materials

In this experiment, experimental materials of different varieties of onions obtained from Melkasa Agricultural Research Center (MARC) found in the Eastern part of Oromia, Ethiopia, were used. The different onion varieties used in this experiment were Adama Red, Nafis, and Monarch varieties.

Currently, in Ethiopia, six onion varieties have been released by the Ethiopian Institute of Agricultural Research, viz. Adama Red, Bombay Red, red creole, Memiru Brown, Memiru White, and Nasik Red varieties. Conversely, no varieties were tested in the study area, and there was also no onion seed supply in the study area. The main reasons and causes behind this still have to be further inspected. In Ethiopia in general and in the western part of the country in particular, the monarch is the most grown variety under rain-fed. Farmers and small-scale producers have a preference for a monarch, mainly because it produces well, i.e., a high yield compared to other varieties ^6^.

However, farmers prefer the monarch due to its first introduction by traders, and no comparison and study was made to recommend the better onion varieties for the research area. Thus, the varieties like Adama Red, Bombay red, and Nafis were not well known in the study area. Adama red variety was allowed in 1980 by the Federal Research Institute of Ethiopia (Melkasa-Agricultural-Research-center). It is finely modified at an altitude of 1600–2800 m above sea level. Nafis variety was released in 2010. It is well adapted to an elevation of 1600–2800 m above sea level. The monarch is a hybrid variety recently introduced to Ethiopia, and its total bulb yield was 32.76 t ha^−1^ (Table 1). The plant we have used in this report was cultivated in the local area of DambiDollo Town, Oromia, Ethiopia ^6^. This study complies with relevant international, national, institutional, and legislative guidelines.

**Table 1:** Released onion varieties in Ethiopia.

Variety	Maturity days	The yield of seed (t ha^−1^)	Bulb’s color	Profit in research field (t ha^−1^)	Yield in farmers' fields (t ha^−1^)
Bombay Red	90–110	1.3–2.0	Light red	25–30	NA
Adama Red	110–130	1.0–1.3	Dark red	30–35	9–15
Red Creole	130–140	0.2–0.6	Red	27–30	9–15
Memiru Brown	120–130	1.2	Brown	NA	NA
Memiru White	NA	NA	White	NA	NA
Nasik Red	100–115	1.4	Slightly reddish	26.5	18.7
Melkam	130–142	1.1–1.5	Red	40	NA
Nafis	90–100	NA	NA	40	NA

Source: Ministry of Agriculture, 2016^9^.

*NA* not available.

### TrialDesign and treatments

The treatments consisted of three varieties of onion (Adama Red, Nafis, and Monarch) and four levels of distance between plants (6 cm, 8 cm, 10 cm, and 12 cm) which were arranged as a complete randomized block design (CRBD) in the factorial arrangement which is duplicated three times. Therefore, a total of 12 combinations of treatments comprised 36 experimental plots, and randomly, each treatment was assigned to the plots. The plot size was 1.8 m by 2 m which is 3.6 m^2^, occupying five rows with 30, 22, 18, and 15 plants on each row for the spacing of 6, 8, 10, and 12 cm, respectively. Each plot consisted of 150 plants for 6 cm, 110 plants for 8 cm, 90 plants for 10 cm, and 75 plants for 12 cm; a distance of 1.0 m was reserved between the blocks, and 0.5 m space was kept between each plot within a block. Exterior lines on both edges of the plots as well as at together edges of row three plants were deliberated (edge plant) to evade border effects.

### Experimental procedures

**Figure 1:**
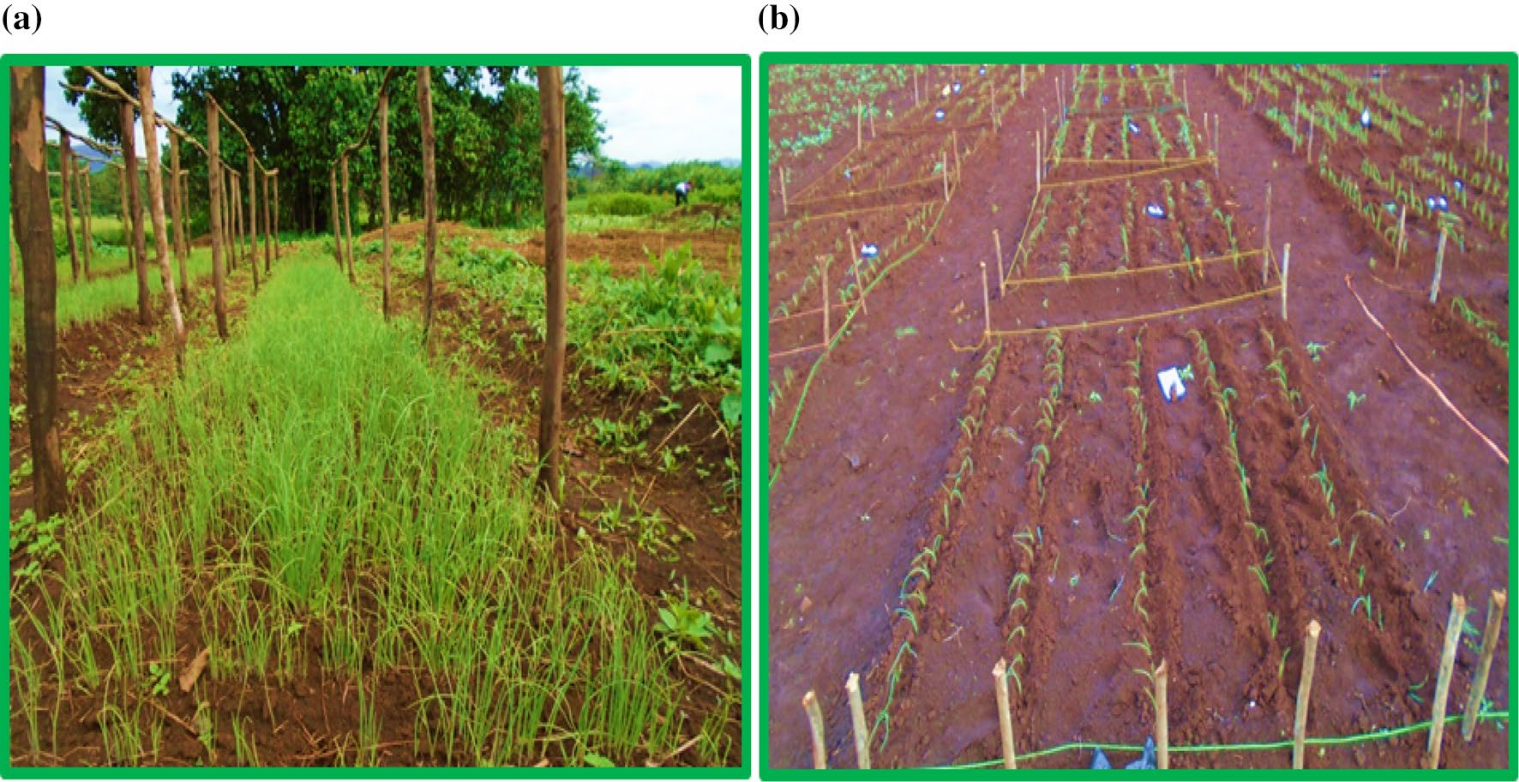
(**a**) Seedlings of onion in the nursery, (**b**) planting of seedlings on the main field.

Well-prepared seed beds were sown with seeds, and weeding at the nursery was carried out by hand. The time of sowing the seeds in the investigational beds was made in the 2nd week of April 2021. Before planting the plantlets, the test field was cultivated, sorted, and weighed well. Transplanting was carried out in the first week of May 2021 when they produced 3–4 true leaves (reached a height of 12–15 cm) by cautiously weeding from the nursery seedbed. The seedlings were watered in a nursery one day before the seedlings were transplanted to make it easier to uproot and maintain a good seedling field during replanting. Healthy and uniform seedlings grown in the middle of the seed beds were used for transplanting. Filling of damaged seedlings is done after seven days to substitute those seedlings that failed to establish after planting. Figure 1 (a) shows Seedlings of Onion in the Nursery and (b) Planting of Seedlings on the main field; other recommended agronomic procedures, such as weed control, crop protection, etc., were kept the same throughout treatment.

### Data collected

#### Phenology

Days to maturity: registered when 90% of the plants in each plant show a collapse of the neck by way of the definite days from transplantation to the time.

#### Growth parameters

Leaf width or diameter (cm): was registered in 10 casually selected plants per plot, and the average of the longest leaves should be sampled.

The number of leaves/ plants: was recorded when matured from 10 plants per plot.

Plant height (cm): The plant height of 10 casually designated plants on each plot is computed by meters and calculated from the bottom to the top of the leave at ripeness.

#### Profit and harvest-related traits

Sample plants between three rows were used to record yields and yield-related data. At physiological maturity, when 90% of peaks fell or the leaves withered, the plants were harvested as well as utilized to determine the yield of bulbs and yield-associated parameters ^10^. Collected bulbs are cured for a week before topping in the shade (screen house).

Bulb diameter (cm): Bulb diameters were calculated from ten casually nominated onions per plot and were measured using a caliper.

Merchantable bulb’s profit (t ha^−1^): was recognized after dumping splatted bulbs, dense necked, unpleasant bulbs, and commercial new bulbs; profits were calculated from the yield of net plots. The merchantable bulbs harvest weight standard in Ethiopia is categorized as extra-large (beyond 160 g), large (100–160 g), medium (50–85 g), and lesser size(21–50 g) ^6^.

Unmarketable bulb yields (t ha^−1^): were decided by classified as: below sized (< 20 g and > 160 g), contaminated, rotten, and disordered physiologically (thick-necked and divided bulbs). These bulbs were weighed and expressed as not commercial bulbs from the net plot area and later extrapolated to a per-hectare basis.

Overall bulb’s harvest (t ha^−1^): The sum of merchantable and marketless bulb yields was computed in tons for each hectare.

### Statistical analysis of data

The composed data were exposed to variance examination (ANOVA) in the overall rectilinear model of the Genstat 16^th^ edition statistical package. The slightestImportant Differences (LSD) at 5% possibility levels were used to associate the conduct rate at which ANOVA showed a significant difference.

## Results and discussion

### Phenology and growth parameters

#### Times to maturity

**Figure 2:**
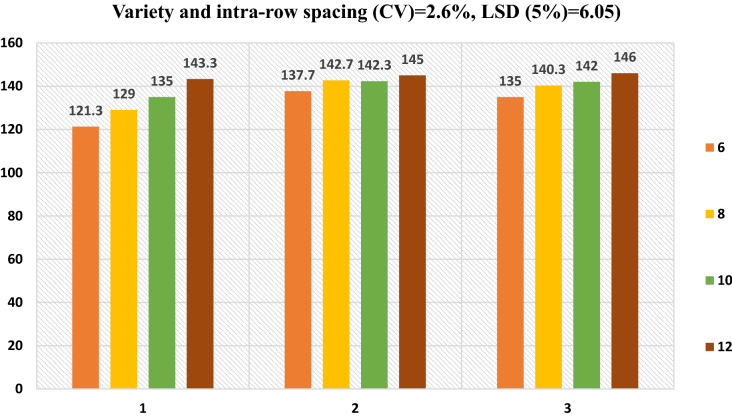
Interaction influences diversity and intra rows space on days to physiological maturity.

Investigation of variances indicated that days to 90% physiological maturity of the onion varieties were greatly affected (P < 0.001) by the variety and intra-rows space, and their interaction significantly (Fig. 2).

Maturity dates fluctuated between 121.30 days and 146.00 days. The Adama red variety reached early physiological maturity (121.3 days) at a narrow space of 6 cm, while the Nafis varieties were lately matured (146 days) at a wider space of 12 cm (Fig. 2).

The prolonged maturity of onions at the broader distance between plants may be recognized to the low competition for nutrients and light in broader spacing, subsequent comfortable leaf development, and delays in the ripening of bulb^2^. Noted the delayed ripening of onions from sporadically occupied plants and plants grown in closer spacing or in high plant spacing ripe early.

#### Plant’s height

Plants height was considerably influenced by varieties and intra-rows, and the collaboration of the two influences does not suggestively upset plant height. Decreased crop height in a wide area may be associated with increased competition for growth resources, leading to poor crop yields. And the maximum plant height (61.60 cm) was recorded in the Nafis onion variety. Similarly, the shortest plant height (54.39) was observed from Adama red onion variety Fig. 2. Likewise^11^, reported Onion Nafisvariety provided the maximum (64.33 cm). Likewise^34^, The highest plant height was obtained in varieties Nafis (10.2 kg).

In another study^12^, documented that the maximum height of plants was observed from the Nafis variety as compared to others. As the distance between plants increases from 7 to 13 cm, the plant height increases. This result is inconsistent with the outcomes of ^13^ who reported that the longest onion plant was recorded at 15 cm between plants, then plants have grown up through 10 and 7.5 cm.

On the other hand^14^, described that the height of the shallot increased because of the increase in intra-rows space at 20 cm positioning caused by the greatly considerable plant height of the shallot. Consistent with current findings^15^, also reported highest onion height was recorded at a 10 cm spacing between plants at Aksum, northern Ethiopia.

#### Number of leaves

The number of leaves was suggestively affected by varieties and intra-rows space, and the collaboration of the varieties and intra-rows-space did not affect this parameter. The highest number of leaves (11.47) were recorded at the spacing of 10 cm between plants which is 46.11% more than the lowermost charged documented at an intra-row space of 6 cm (Fig. 2).

In another way, the highest number (10.55) was recorded in the Nafis variety, and the plants with the lowest number of leaves (8.840) were recorded in Adama red variety (Fig. 2). Likewise^16^, reported that the highest number of leaves were observed in wider plant space^17^. Also showed that as plant population density decreases, the number of leaves produced per plant increases.

#### Leaf length

**Figure 3:**
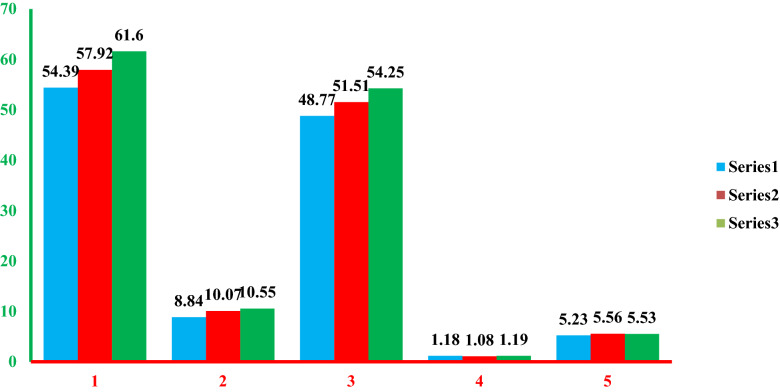
The main influence of Varieties and intra-row-space on plants length, leaf number, leaf length, leaf's diameters, and bulb diameters of onion varieties. *PH* plants length in cm, *LN* leaf's numbers, *LL* leaf's length in cm, *LD* leafs diameters, *BD* bulb’s diameters, means sharing similar letter(s) are not meaningfully dissimilar at p < 5% bestowing to LSD (least significance difference) test.

Leaf length was suggestively influenced by the foremost effect of plant spacing and varieties. The highest leaf length (54.25 cm) is found in the Nafis variety, and the lowest leaf length comes from Adama red variety (48.77 cm) (Fig. 3). The alteration in the length of a leaf between varieties may be due to their genetic differences. Likewise^18^, showed that the highest length of leaf (43.24 cm) from the onion varieties of Nasik Red was linked to other varieties.

The length of the leaf increases as the space between plants is prolonged from 6 to 12 cm. The longest leaves are seen on plants separated at a distance of 10 cm (54.01 cm) and 12 cm (52.54 cm) distance between plants. Although, the plants spaced at 6 cm (49.20 cm) and 8 cm (50.29 cm) showed the minimum length of leaf in Fig. 3. This may be recognized as the accessibility of extra nutrients and moistness in the wide area between the plants, while the closer distance between plants tends to strong nutrients and moisture competition and thus cause the plant to be shorter^19,20^. Reported alike results when the highest leaf length was found in the wide space between plants.

The increase in leaf length of onion varieties in a wide area may be due to suitable distance between plants that allows the leaf to grow robustly and, as a result hence, enhances plant growth. This result is inconsistent with the results of ^15^ who documented that the highest onion leaf length was found to be 10 cm distance between plants than plants arranged at 5 cm as well as7.5 cm plant space at Aksum, northern Ethiopia. In addition, many researchers have shown that a higher length of a leaf was observed in plants set apart at a broader arrangement ^21–23^.

#### Leaf diameter

Leaf diameter was greatly influenced by intra-rows-space and varieties, whereas the interactions did not show significant results. Increasing the spacing between plants from 6 to 12 cm increases the diameter of the leaf from 1.12 to 1.20 cm (Fig. 3). Likewise^11^, reported that as the distance between plants increased, the diameter of the leaf increased.

The width of the broad leaf produced in the wide area of intra-row may be due to the isolated plants receiving the right amount of light that is more imperative for photosynthesis and nutrients associated with distant plants. The highest leaf width is found in plants planted in a wide area between rows compared to a plant planted in close proximity.

#### Bulb diameter

Bulb diameters of onion plants are strongly affected by the main influence of diversity as well as spacing between plants (P ˂ 0.001), and the interaction of these two factors did not significantly affect bulb diameter.

Variety monarch provided the maximum bulb diameters (5.56 cm). The largest bulb’s diameter (5.50 cm) is found in onion plants planted at a 12 cm distance between plants, followed by plants planted at 8 cm between plants (5.47 cm). Plants separated by 6 cm intra-row provided the lowest bulb diameter (5.33 cm). The plants grown at a spacing of 10 and 12 cm does not show substantial variation between plants grown at 10 and 12 cm.

The increment of bulb diameter in a wide plant spacing may be due to the availability of reserved food nutrients, moisture accessibility, and causing an expansion in bulb size. Likewise, a great plant population indicates the nearer proximity and final decrease of available area per plant, and then it is true that enlargement of the bulb may be inadequate due to reduced planting space ^24^. The current findings are consistent with ^25^ who obtained the maximum bulb diameter from wider intra-row spacing). Likewise^11^, reported that the maximum diameter of the bulb (5.63 cm) was recorded at a 13 cm distance between plants.

### Yield and yield components

#### Marketable yield

Marketable bulb’s yields were suggestively (P ˂ 0.001) affected by variety as well as space, while the interface didn’t influence marketable bulb yield. The Nafis varieties have yielded the uppermost commercial bulb’s yield (34.44 t ha^−1^), followed by varieties monarch (31.45 t ha^−1^), and the lowermost commercial bulb’s yields (21.74 t ha^−1^) were gained from varieties of Adama red (Table 2). Likewise^34^, the highest marketable bulb yield per plot, was attained in diversities Nafis (10.2 kg).

**Table 2:** Effect of varieties and space on merchantable yields, non-marketable bulbs yields, and total bulb yields of onion varieties.

Varieties	Treatment		
	Merchantable bulbs yields (t ha^−1^)	Non-marketable bulb yields (t ha^−1^)	Total bulb yields (t ha^−1^)
Adama red	21.74^c^	1.18^a^	22.92^c^
Monarch	31.45^b^	1.31^a^	32.76^b^
Nafis	34.44^a^	0.96^b^	35.40^a^
LSD (5%)	0.74	0.31	0.77
**Intra-row spacing (cm)**			
6	27.17^c^	1.93^a^	29.10^c^
8	29.02^b^	1.33^b^	30.35^b^
10	31.12^a^	0.66^c^	31.78^a^
12	29.52^b^	0.68^a^	30.20^b^
CV (%)	3.0	32.5	3.00
LSD (5%)	0.85	0.36	0.89

Mean within a pole followed by the same letter(s) are not meaningfully dissimilar at p < 5% according to LSD (least significance difference) test.

The differences observed between onion varieties may be attributed to the ability to work under different agricultural climatic conditions and genetic makeup. Consistent with the present result^12^, stated the higher and lower yield of the bulb (15.94 t ha^−1^ and 9.17 t ha^−1^) from diversityNafisas well as Adama Red separately. The highest commercial yield of the bulb (31.12 t ha^−1^) was recorded on 10 cm planted crops, and the lowermost saleable bulb yield (27.17 t ha^−1^) was observed on 6 cm planted crops Table 2.

The size of the bulb under a wider space did not recompense for the decrease in yield per hectare initiated by a decrease in the number of plants in the wider space ^29^. Plant density affects the marketable bulb yield, and the high plant population results in reduced marketable bulb size. The higher yields of commercial yields in the smallest distance between plants may be owed to more plant density, producing more bulb yields^26^. Obtained the uppermost commercial yields (34.49 t ha^−1^) from the nearby space (5 cm). Russo (2008) also showed that 97% of marketable bulbs of onion are from a closely occupied plant. The result is inconsistent with those of ^16^ who documented that surpassed bulbs of *Huruta*shallot implanted at a 10 cm distance between plants gave the highest yield per hectare compared to 15 and 20 cm spacing^15^. Likewise stated, an increase in the distance between plants from 5 to 10 cm reduced merchantable bulb’s yields from 34.49 to 28.10 t ha^−1^^19^. Also found a reduced yield of marketable bulb yield as the space between the plant's increases from 5 to 10 cm. In contrast, the yield of shallot was high at broader intra-row-space (20 and 25 cm) as compared to 15 cm spacing ^27^.

#### Unmarketable bulb’s yields

Unmarketable bulb’s yields were significantly (P < 0.001) affected by variety and intra-row- space, while the interaction did not affect unmarketable bulb yield. The maximum yield of an unmarketable bulb (1.93 t ha^−1^) was observed on a plant planted in a narrow space (6 cm) and wider (12 cm) spacing (Table 2).

The highest unmarketable yield in a narrow space may be related to increased rivalry for growing assets and a lack of nutrients for the progress and expansion of all plants in the stand. The lesser non-marketable yields in greater spacing may be due to the limited opposition for growth resources leading to large segments that increase the yield of the unmarketable bulb.

This may be particularly noted in the case of nitrogen deficiency and poor plant growth, leading to weaken plants' susceptibility to other biotic and abiotic pressure and assimilate production, leading to reduced bulb size ^21–23^. Also showed that the control treatment yielded a high unmarketable yield, while low non-marketable yields were related to high nitrogen levels.

The highest non-marketable yields in the closest spacing may be due to competition between plants and growth-promoting resources, which has led to a much lesser level of big bulbs than wider spacing. In addition, it may be due to poor nutrition, light, and space in dense vegetation. The results are concomitant with ^9^ and ^16^, who noted that the space between the narrow spacing increases the yield of unmarketable bulbs of onion and shallot, respectively.In contrast^28^, documented that wider spacing yielded maximum unmarketable yield due to higher bulb diameter that resulted in an oversize bulb and consequently higher unmarketable yield.

#### Total bulb yield

The total bulb’s yields were expressively (P ˂ 0.001) affected by varieties as well as intra-row -spaces, while the influence of the interaction was not. The maximum yields of bulbs (35.40 t ha^−1^) are attained from the Nafisvariety followed by a monarch (32.76 t ha^−1^), while the lowermost bulb yield (22.92 t ha^−1^) was observed in the diversity Adama Red (Table 2).

Differences in the production of bulbs are contingent on the difference in cultivars, agroecology, agronomic applications, and their relations. The outcome is concomitant with the findings of ^12^ who initiated Nafis variety gave the maximum total bulb yield than other varieties.

In addition, the maximum amount of marketable yield (31.78 t ha^−1^) and the minimum commercial yield (29.10 t ha^−1^) was attained in the closest (6 cm) and widest (10 cm) distance between plants correspondingly. The increase in the total yields of bulbs by a high number of plants may be due to the increase in plant stand and, as a result, the advanced bulb number formed in each area. Conversely, the weights of the bulb are decreased because of high opposition between plants for development influences. Likewise^25^, reported higher yields of total bulbs from onion crops that have more populations than those planted less.

Therefore, using a 10 cm spacing is an excellent dose of onion for commercial and produces a better bulb yield. Similarly, Refs.^29–35^ reported that an increase in bulb yield is due to increased nitrogen consumption. Al-Frahat (2009) also pointed out that the 0 NPS applied plots recorded inferior yield as associated with the greater nitrogen dose. Likewise^14^, reported that the Nitrogen level increase from 100 to 150 kg ha^−1^ didn’t raise yields.

Moreover, this finding is similar to the discoveries of ^36–39^, who described that proliferation in the distance between shallots plants from 5 to 20 cm occasioned a decrease in yields from 36.0 t ha^−1^ to 23.9 t ha^−1^ but most (86%) of the bulbs was very low and therefore could not be unmarketable at 5 cm intra-rows- space. Likewise^16^, found that the amount of total yield reduced with the rise in the intra-row-space of shallot.

## Conclusion and recommendations

In conclusion, the major impact of the variety of onions and intra-row spacing greatly affects all phenological growth variables and yields contributing traits of onion crops. The variance of analysis displayed that except for days to 90% physiological mellowness, which is affected by the interactions influences of variety and intra-rows-spacing, altogether, constraints are greatly affected by the variety and distance between plants.

From this finding, the highest yield of the marketable bulb (31.12 t ha^−1^) of onion was recorded on 10 cm planted crops for intra-raw spacing, and low yields of the marketable bulb (27.17 t ha^−1^) are noted on plants planted at 6 cm intra row spacing. Additionally, the maximum commercial yield (34.44 t ha^−1^) was noted from the variety Nafis, and the lowest value (21.74 t ha^−1^) was documented from Adama red variety. Considering the main influences of space, the maximum yield of an unmarketable bulb (1.93 t ha^−1^) was documented in plants with a small intra-raw space, and the lowest value (0.66 t ha^−1^) was documented in plants with 10 cm intra-raw-space. And the maximum non-marketable yield (1.18 t ha^−1^) was noted from Adama red variety, and the lowermost unmarketable yield (0.96 t ha^−1^) was observed from the Nafis varieties. Thus, it is conceivable to decide that 10 cm intra-row-spacing, as well as the varieties of Nafis, will be used for the production of onions at the research site. Nevertheless, this study was conducted only one season in one place; therefore, the same research should be done under different agro-climate and soil conditions in order to make a complete recommendation.

## Data Availability

The datasets used and analyzed during the current study are available from the corresponding author on request.
